# A Multimodal Hydrogel Soft-Robotic Sensor for Multi-Functional Perception

**DOI:** 10.3389/frobt.2021.692754

**Published:** 2021-08-26

**Authors:** Yu Cheng, Runzhi Zhang, Wenpei Zhu, Hua Zhong, Sicong Liu, Juan Yi, Liyang Shao, Wenping Wang, James Lam, Zheng Wang

**Affiliations:** ^1^ Shenzhen Key Laboratory of Biomimetic Robotics and Intelligent Systems, Department of Mechanical and Energy Engineering, Southern University of Science and Technology, Shenzhen, China; ^2^ Guangdong Provincial Key Laboratory of Human Augmentation and Rehabilitation Robotics in Universities, Southern University of Science and Technology, Shenzhen, China; ^3^ Department of Mechanical and Energy Engineering, Southern University of Science and Technology, Shenzhen, China; ^4^ School of Innovation and Entrepreneurship, Southern University of Science and Technology, Shenzhen, China; ^5^ Department of Mechanical Engineering, The University of Hong Kong, Hong Kong, China; ^6^ Department of Computer Science, The University of Hong Kong, Hong Kong, China

**Keywords:** soft sensor, multimodal, multifunctional perception, hydrogel, electrical and optical properties

## Abstract

Soft robots, with their unique and outstanding capabilities of environmental conformation, natural sealing against elements, as well as being insensitive to magnetic/electrical effects, are ideal candidates for extreme environment applications. However, sensing for soft robots in such harsh conditions would still be challenging, especially under large temperature change and complex, large deformations. Existing soft sensing approaches using liquid-metal medium compromise between large deformation and environmental robustness, limiting their real-world applicability. In this work, we propose a multimodal solid-state soft sensor using hydrogel and silicone. By exploiting the conductance and transparency of hydrogel, we could deploy both optical and resistive sensing in one sensing component. This novel combination enables us to benefit from the *in-situ* measurement discrepancies between the optical and electrical signal, to extract multifunctional measurements. Following this approach, prototype solid-state soft sensors were designed and fabricated, a dedicated neural network was built to extract the sensory information. Stretching and twisting were measured using the same sensor even at large deformations. In addition, exploiting the distinctive responses against temperature change, we could estimate environmental temperatures simultaneously. Results are promising for the proposed solid-state multimodal approach of soft sensors for multifunctional perception under extreme conditions.

## Introduction

Soft robotics is receiving increasing attention among the researchers and becoming ubiquitous in various fields, including manipulation, human–robot interaction, medical devices, wearable exoskeleton, etc., for its intrinsic compliance, safety, and adaptive continuum deformation (S. Liu et al., 2021; Gifari et al., 2019). Considering the applications of the soft robots accompanies with extreme environments and conditions hazardous for humans, challenges for robots’ safety and reliability are posed by the critical demands. Such extreme application scenarios could include large, coupled, and complex deformation interacting with humans, varying ambient temperature and pressure in aquatic field, or severe environments demanding a high swapping rate of the soft units (Chen et al., 2020). Therefore, the capability of robots to obtain sufficient information from itself and the environment is of non-negligible significance for the implementation of the soft robot. Meanwhile, the perception system would be most desirable with high compactness, without increasing the bulkiness of the robot. To meet the crucial demands in the extreme environment, a sensing system that is able to achieve sufficient proprioception and accommodate to severe environments should be explored and developed.

With reference to the crucial demand of such a sensing unit for soft robotic application, researchers have done fruitful work on soft sensors made of elastic material, achieving easy embedding with the soft actuators and deformation perception (Shen et al., 2021; S.; Zhang et al., 2021). State-of-the-art soft sensor mechanisms mainly adopt two methods to obtain desired information, namely detections of electrical and optical properties. For electrical ones, the resistance of material used in the sensor could change with its geometrical parameters (Chossat et al., 2013; Yiming et al., 2021). Measuring such resistance during deformation reflects the changes in volume or shape. Materials with resistive characteristics include ionic liquids, nanomaterials, hydrogels, etc. (S. Kim et al., 2019; Yiming et al., 2021; Z. Wang et al., 2019; J. Liu et al., 2020; Lu et al., 2019; Yang and Suo 2018). In addition, special alignment of conductive soft layers utilizes capacitance principle to detect deformation of the soft mechanism (Larson et al., 2016). Different from the electrical-property mechanism, the optical ones reversely utilize the fiber-optic for sensing, which detects the light intensity loss in a channel (Shen et al., 2021; T.; Kim et al., 2020; Larson et al., 2016; H.; Zhao et al., 2016). The material filled in the channel of the sensor has a different reflective rate with the channel coating, leading to a detectable light intensity loss during deformation. Besides, other techniques, such as optical wavelength detection, conductive fabric, etc., contribute to the soft sensor field significantly (Bai et al., 2020). However, the existing solutions with single sensing method could neither respond to all the deformation modes, namely the bending, compression, stretching, and twisting, nor differentiate the type of deformation from each other (S. Kim et al., 2019). Furthermore, the working conditions of the soft sensors are restricted due to the sensing material’s properties. For example, ionic liquid remains effective under room temperature, while the moisture loss of the liquid causes malfunction. Therefore, the limitations of the soft sensor: 1) not capable of sensing multiple deformation modes with single sensing method; 2) restricted information collecting ability in harsh conditions; should be further tackled.

**FIGURE 1:**
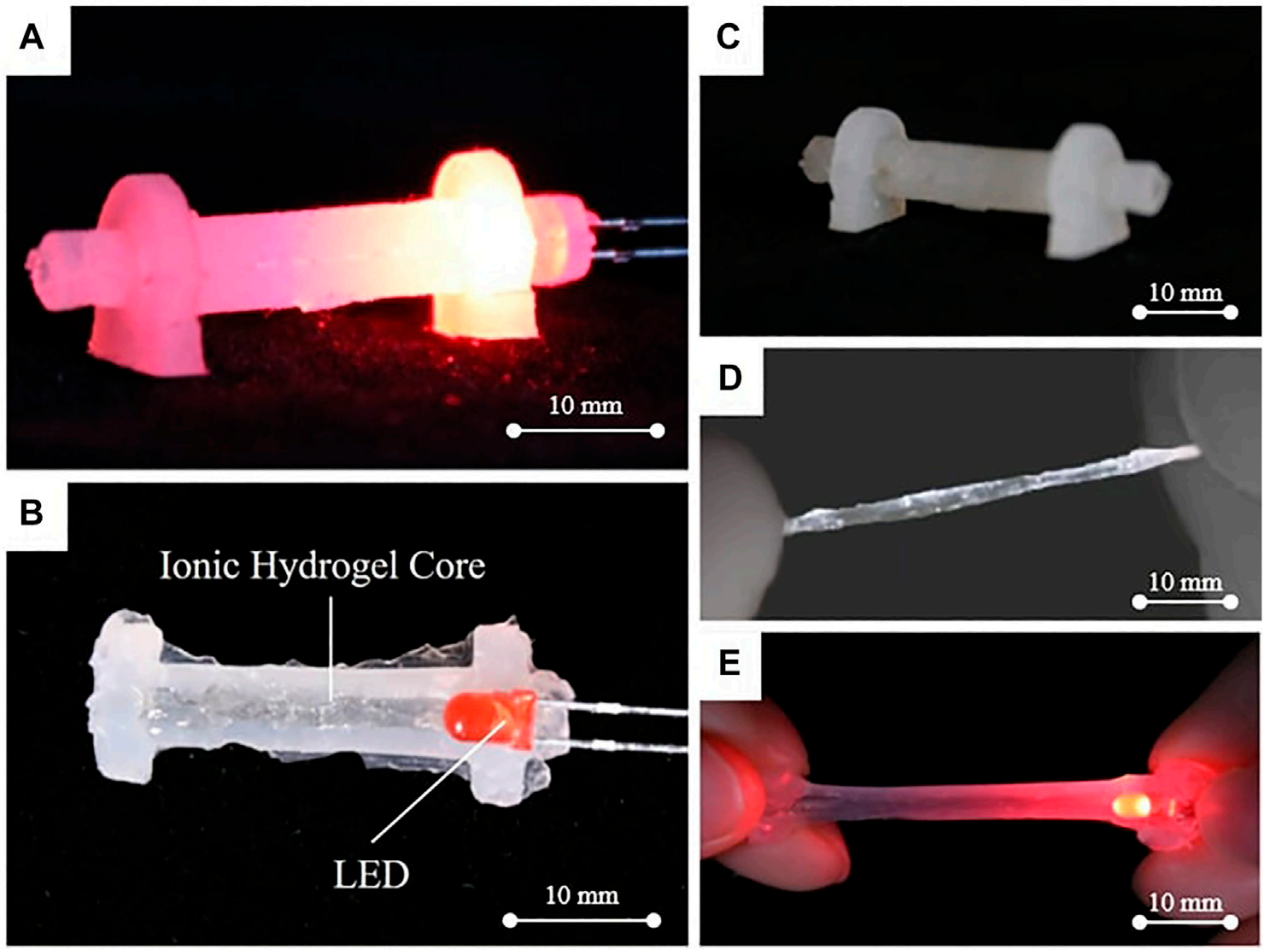
The proposed MHS Sensor. **(A)** The fabricated MHS Sensor with LED operated. **(B)** The cross section of the MHS Sensor. **(C)** The silicone cladding. **(D)** The solidified hydrogel with high intrinsic transparency. **(E)** The MHS Sensor with high stretchable ability.

In this article, we propose a novel solid-state soft-robotic sensor to perceive various modes of deformation, to be effective and robust under severe ambient factors, and to enable the environmental temperature estimation beyond self-state sensing. The proposed multimodal soft-robotic sensor (MHS Sensor), as shown in Figure 1A, could achieve the prodigious features of multifunctional perception with proper design, utilizing standard silicone molding (Figure 1C) and encapsulating techniques (R. Zhang et al., 2020). The proposed sensor consists of novel deployment of hydrogel (Figures 1B,D), integrating resistance and optical intensity detection. The heterogenous sensing (including stretching and twisting) coming with the novel design could be functional under harsh conditions, which are large deformation (within 150% of strain, shown in Figure 1E) as well as low and high temperatures (0–60°C) (when subjected to large deformation and temperature change, namely, deformation of 150% of strain as shown in Figure 1E, and temperature ranging from 0 to 60°C). In addition to the deformation sensing, the two integrated sensing mechanisms enable ambient temperature estimation.

In this work, investigation on the sensing performance in terms of the resistance changing, optical intensity loss under different deformation is conducted respectively. Temperature estimation is also validated in an incubator. The pattern recognition for deformation classification is achieved by artificial neural network (ANN) for real-time deformation perception. The novel design and experimental results of the MHS sensor are presented and discussed in detail to validate the investigations.

## Design and Modeling

### Design Requirements

Numerous extreme application scenarios in soft robotics field have shown the critical demand of a multifunctional integrated sensor unit for proprioception of the large continuum and multi-degree-of-freedom deformation, with ambient environmental information perception, such as the aquatic field with varying temperatures, etc. Apart from the stretching, twisting movements of the robots were shown to be difficult to measure, which is very common in robotic hand or joint motions (S. Liu et al., 2020; Su et al., 2020). The coupled deformation requires different sensing mechanisms leading to system bulkiness and complexity. To tackle the limitations, the proposed sensing unit should be designed with the following desired features:

1) Large deformation range. The sensing unit should be able to conform to large, continuum deformation, such as stretching and twisting. Therefore, conventional rigid mechanism should be eliminated, and sensing mechanism with soft material is preferred for its compliance and elasticity.
2) Multiple sensing principles. To broaden the limited functionality of one simple sensing principle, the sensing unit should be able to integrate multiple sensing principles for utilizing different sensing properties, such as electrical and optical properties. Innovative measures are required to ensure the different mechanisms being able to coexist and coordinate properly within the integrated sensing modular without sacrificing the compactness.
3) Multifunctional perception. With a compact form factor, the sensing system would be desirable to sense multiple types of stimulus, such as different deformations or thermal stimulus. The remarkable ability of both proprio and environmental perception within the compact unit would grant the sensor with wide application potential.
4) Embedment and customization. As a modularized and integrated sensing unit, it should be encapsulated individually via standard fabrication techniques. In addition, the sensing unit should be size-customizable and easy to embed in the robots for different application scenarios.

### Design and Modeling of the Soft Sensor

**FIGURE 2:**
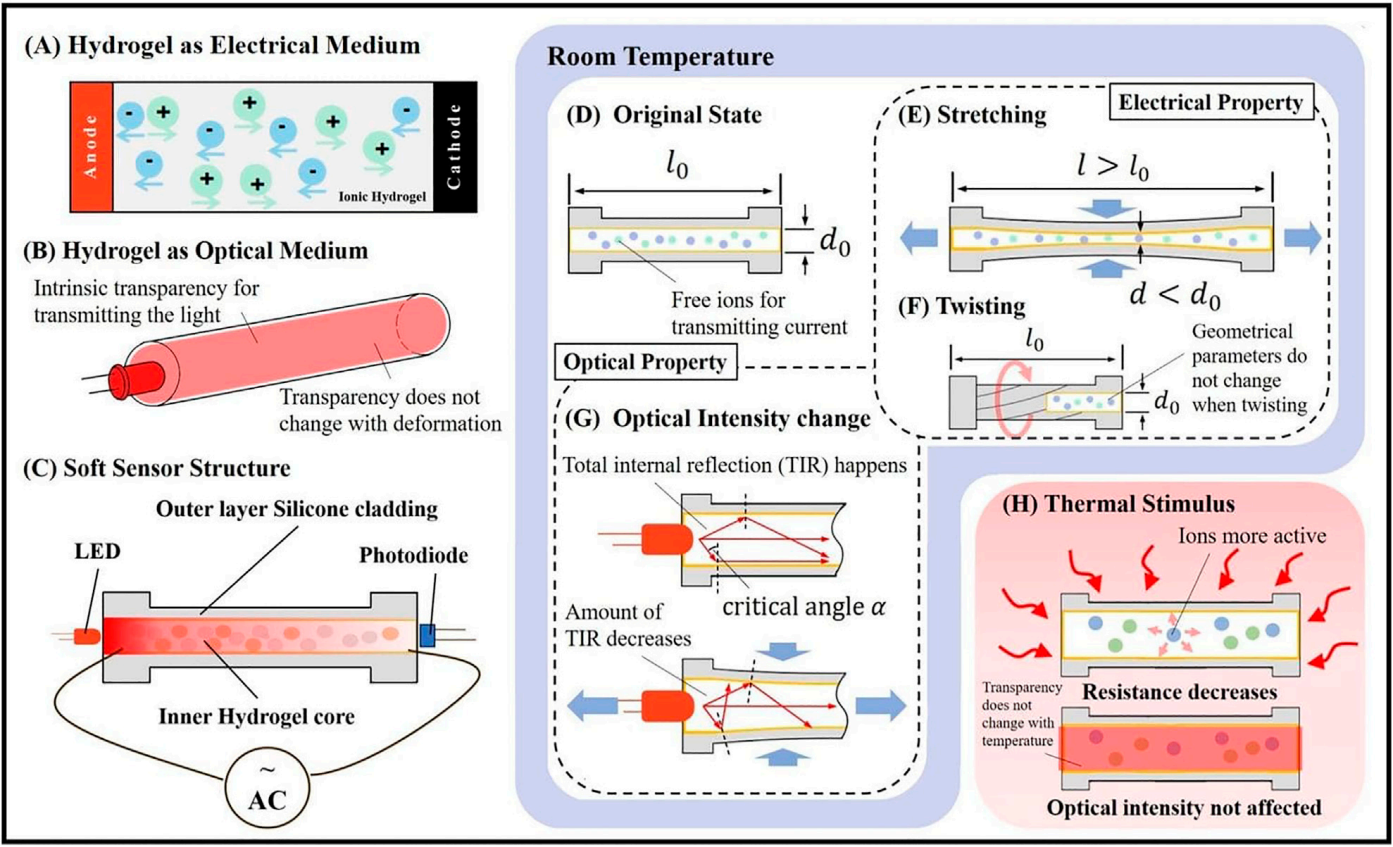
Electrical and optical properties of ionic hydrogel. **(A)** Ions in hydrogel make it a conductive material and resistively detectable. **(B)** The intrinsic transparency of hydrogel enables light to transmit through the core, which does not change with deformation. **(C)** The structure of the MHS Sensor. LED and photodiode are at the two distal ends of the cylindrical silicone cladding respectively. The resistance of the hydrogel core and the optical intensity received by the photodiode are measured to reflect the deformation. **(D)** The original state of the MHS Sensor. **(E)** The resistance of the hydrogel core will increase with the length rising and diameter decreasing when being stretched. **(F)** The resistance will not change when being twisted, as the change in geometrical parameters of the hydrogel core is negligible. **(G)** The received optical intensity can be detected changing, as the amount of the total internal reflection will decrease during deformation. **(H)** Under thermal stimulus, free ions of the hydrogel will be more active leading to resistance decreasing, while the transparency will not change, which means that temperature does not affect the optical intensity received.

To achieve the multifunctional perception of large temperature change and complex, large deformations, the MHS Sensor with elastic cladding and multifunctional core medium is proposed, as shown in Figure 2. Such design benefits from the electrical and optical properties of the hydrogel, achieving three functional perceptions, including stretching deformation, twisting deformation, and temperature estimation. The hydrogel is the material: 1) whose resistance could be changed with respect to the shape deformation and ambient temperature; 2) which can be the medium of the light and has the proper refractive rate for the total internal reflection.

As the electrical medium, free ions residing in the hydrogel help the current transmitting through the material for resistance detecting, as shown in Figure 2A. As the optical medium, the intrinsic transparency of the hydrogel negligibly affects the light transmission, as shown in Figure 2B. To contain the hydrogel core, outer cladding was designed as silicone for its conformation to large deformation. In addition, the elasticity and the geometrical parameters could be customized according to the actual demands of the application scenarios. LED and photodiode form the light path inside the hydrogel channel and Figure 2C shows the entire MHS Sensor’s structure.

To achieve the stretching and twisting sensing, the two properties are utilized, which are resistance and optical intensity. When being stretched, the change of the geometrical parameters of the hydrogel core causes the resistive variation (Figure 2E), compared with the original state (Figure 2D). While being twisted, no resistive change happens since the change in geometrical parameters is negligible (Figure 2F). In terms of the optical intensity sensing mechanism, the MHS Sensor utilizes the principle of total internal reflection. Once the angle of incidence of the light is larger than the critical angle that is determined by the refractive rate of the core and the cladding (X. Wang et al., 2015), the total internal reflection would happen (Figure 2G-upper). The amount of the total internal reflection would be affected by the deformation of the soft sensor, leading to detectable signal change for sensing (Figure 2G-below).

The different responses of the electrical and optical properties under thermal stimulus grant the MHS Sensor with potentials to work under a range of temperatures and estimate the ambient temperature as shown in Figure 2H. With the temperature rising, the free ions in the hydrogel become more active, enhancing the current transmission through the material. Therefore, the resistance of the hydrogel would decrease when being heated, while the transparency of the hydrogel would not vary with the heat, leading to temperature-insensitivity of the optical transmission. Through this difference, sensing under different temperatures could be achieved by the compensation of two properties and the environmental temperature estimation could be realized.

Overall, the proposed combination of the silicone cladding and the hydrogel core could fulfill the requirements of the soft sensor with the electrical and optical signal varying respectively with the deformation. Detecting and processing such two signals could recognize the mode and the magnitude of the deformation. The thermal characteristics of the proposed sensor would bring benefits of environmental perception.

For a cylindrical shape hydrogel with length L, cross-sectional area S, and electrical resistivity ρ, the resistance of the conductive hydrogel could be expressed as

$$R=\rho \frac{L}{S}$$ (1)

For the ionic hydrogel, the relationship between the conductivity σ and electrical resistivity ρ is

$$\sigma =\frac{1}{\rho }$$ (2)

According to the Arrhenius equation and Vogel–Fulcher–Tammann (VFM) equation, the temperature dependence of any thermal activation process can be described as

$$\sigma =\frac{\sigma_{0}}{T}\text{exp}\left(-\frac{E_{A}}{RT}\right)$$ (3)

where the $\sigma_{0}$ and $E_{A}$ may itself be a weak function of temperature [(Q. Zhao et al., 2020)],

$$R=\rho \frac{L}{S}=\frac{1}{\frac{\sigma_{0}}{T}\text{exp}\left(-\frac{E_{A}}{RT}\right)}\frac{L}{S}$$ (4)

Thus, the relationship of the core’s resistance and the geometrical parameters as well as the ambient temperature could be derived. Through the relation equation, the deformation could be estimated in terms of the mode and the magnitude.

Apart from being the conductive medium for detecting the resistance changing, hydrogel could be the optical medium for its inherent transparency. The total internal reflection of certain wavelengths would happen through the core hydrogel and the photodiode could sense the optical intensity at the distal end of the MHS Sensor. Such optical intensity would vary with the amount of the total internal reflection due to deformation. The optical intensity transmitted to the distal end through the hydrogel could be expressed as [(H. Zhao et al., 2016)],

$$A=ecL=log_{10}\left(\frac{I_{0}}{I}\right)+b$$ (5)

$$log_{10}\left(\frac{I_{0}}{I}\right)=ecL-b$$ (6)

where A is absorbance, L is the hydrogel length, e is the absorptivity of transparent hydrogel, c is the concentration of the material absorbing the light in the hydrogel, b is the baseline absorbance. e and b are constant which means there is a nonlinear relationship between I and L. When the length L of the hydrogel increases, optical intensity I decreases.

By analyzing the two signal modalities generated by the MHS Sensor under different deformation, the deformation type and magnitude can be obtained. Furthermore, the two properties of the hydrogel (electrical and optical) could derive additional perception, i.e., the ambient temperature estimation. When subjected to a thermal stimulus, the resistance of the hydrogel changes, while the transparency and the refractive rate stay unaffected. Thus, the optical intensity transmitted to the end of the sensor would not vary with the temperature. When subjected to a deformation with a temperature higher/lower than the room temperature, the resistance of the sensor is affected by both deformation and temperature. By using the optical information as a reference, the temperature influence can be decoupled from the deformation; thus, the ambient temperature could be estimated. In this way, the multifunctional perception (stretching, twisting and ambient temperature) can be realized by the compact design of the sensor, consisting of silicone cladding and hydrogel core.

### Signal Processing Scheme

**FIGURE 3:**
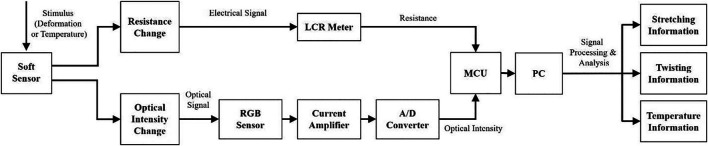
The signals collection and processing scheme of the soft sensor.

To analyze the electrical and optical signal generated from the sensor’s deformation or environmental stimulus, a collecting and processing system has been designed, as shown as Figure 3. For resistance measurement, the LCR is adopted for avoiding electrolysis of ion hydrogel and ensuring the accuracy and stability. The RGB sensor is adopted to detect the optical intensity transmitted through the hydrogel core from the LED in the other end. Current would be generated by the RGB sensor according to the variation of the optical intensity. Then *via* current amplifier and digital converter, the signal is outputted to the MCU connected to the PC. Thus, the direct collection of the two signals information could be processed by the PC and further analysis on the deformation and environmental factors modes could be done.

## Fabrication and Experiments

To validate the deformation sensing of the MHS Sensor, prototypes were fabricated and tested. An appropriative testing platform was set up for stretching and twisting tests. Results and discussion are presented in detail.

### Fabrication of the MHS Sensor

**FIGURE 4:**
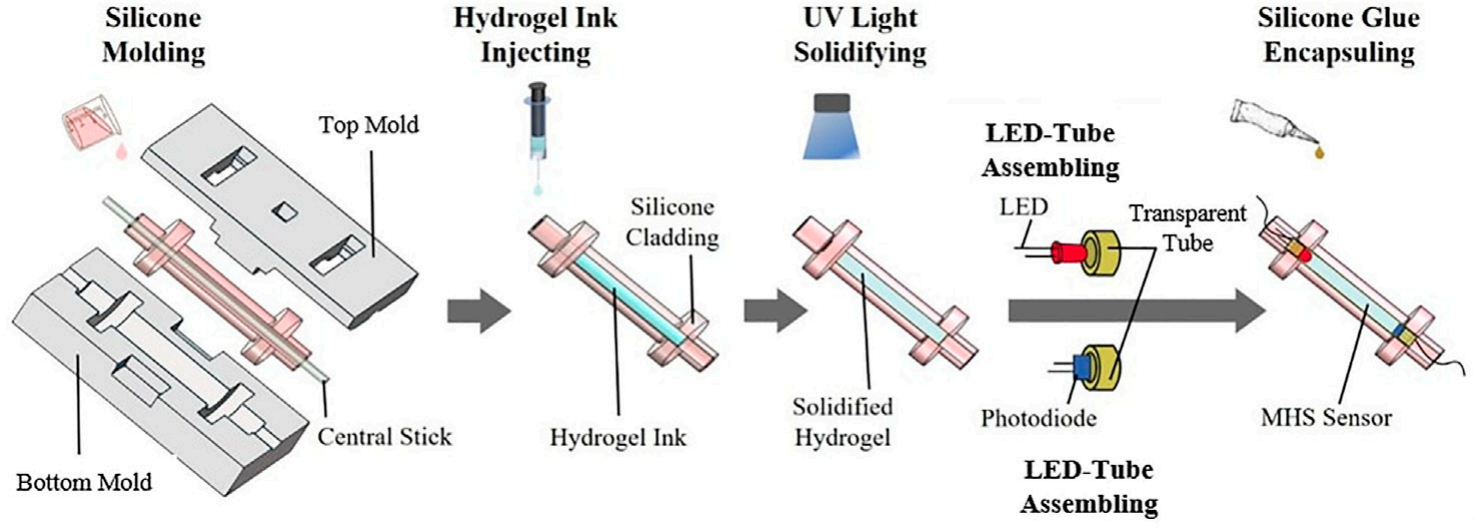
Procedures for fabricating the MHS Sensor.

The proposed solid-state MHS Sensor consists of three parts: the elastic cladding, the hydrogel core, and the encapsuling ends. The detailed fabrication procedures are illustrated in Figure 4. The cladding was made of silicone by molding. The design of the sensor takes advantage of the resistance change and optical intensity loss caused by deformation. The inner core cavity was filled with hydrogel; a light-emitting diode (LED) was fixed at one end, an RGB sensor for measuring optical intensity at the other end. To assemble such three parts into one sensing unit, silicone cladding (ecoflex-0030, Smooth-On) was first casted to make a cylindrical cavity. The hydrogel precursor ink (PAMM-LICl) was injected into the cavity both for detecting resistance and as the medium for light (Gu et al., 2019). Then the hydrogel was exposed under the UV light for solidifying. The solidified hydrogel is with a refractive rate of 1.38, which is higher than that of the silicone cladding (1.35), facilitating the total internal reflection of the light (refractive rate measured by ATAGO—PR-RI (1.3006–1.4436) refractometer). LED and the RGB sensor together with a transparent tube with a conductive wire were then inserted into the solidified hydrogel at the two ends respectively and fixed by silicone glue. The glue encapsuling is to prevent the hydrogel from evaporating during deformation. Overall, three MHS Sensors with different diameters were fabricated with a cylindrical cladding thickness of 1 mm and the inner channel of 20 × 2 mm, 20 × 3 mm, and 20 × 4 mm.

### Experimental Apparatus

**FIGURE 5:**
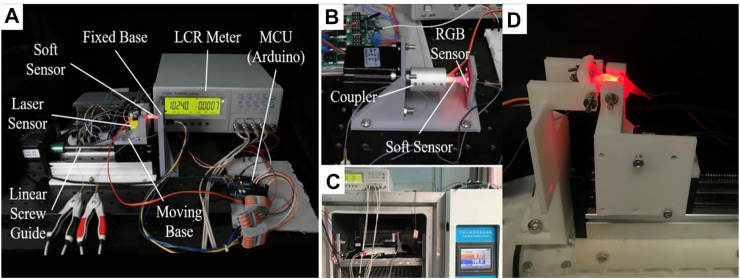
Experimental apparatus. **(A)** Equipment for stretching test of the soft sensor. **(B)** Equipment for twisting test of the soft sensor. **(C)** The incubator for temperature test. **(D)** Equipment for bending test of the soft sensor.

To investigate the electrical and optical behavior of the MHS Sensor under different deformation modes, experimental apparatuses for stretching and twisting test respectively were developed, as shown in Figure 5. For stretching test platform (Figure 5A), the stepper motor with a guide-screw was adopted to generate a linear motion. The two ends of the soft sensor were installed on the 3-D printed components connected to the sliding part on the guide. Thus, the motor controlled the soft sensor to stretch and contract at a constant speed along its axis. In addition, the displacement of the soft sensor was measured by a CMOS type Micro Laser Distance Sensor (Panasonic HGC1050, 50 mm, 30 μm resolution) for obtaining the relationship of the resistance and the strain. As for the twisting test (Figure 5B) the stepper motor was also adopted without the guide-screw. Therefore, the motor rotation directly twists the soft sensor around its axis. Likewise, the installation components were fabricated by 3-D printing techniques. In this case, the twisting angle was not directly measured; instead it was calculated by the steps of the motor.

Furthermore, to investigate the MHS Sensor’s sensibility under a range of ambient temperatures, an incubator (LY-2150) was utilized to conduct resistance and optical intensity-strain relation tests under different constant ambient temperatures, as shown in Figure 5C. The stepper motor and soft sensor were installed inside the incubator, while the signal detecting and processing system were at outside for electronics safety. Figure 5D shows the bending apparatus for the proposed sensor, the stepper motor will move to control the distance of the two ends of the MHS sensor. Each end of the sensor was connected to a freely rotating hinge, respectively, to ensure the proper bending gesture.

With the three sets of the experimental apparatus, the relationships of resistance and optical intensity and the strain and twisting angle could be investigated for further analysis on the deformation type, magnitude, and the temperature characteristics of the MHS Sensor.

### Stretching Tests

**FIGURE 6:**
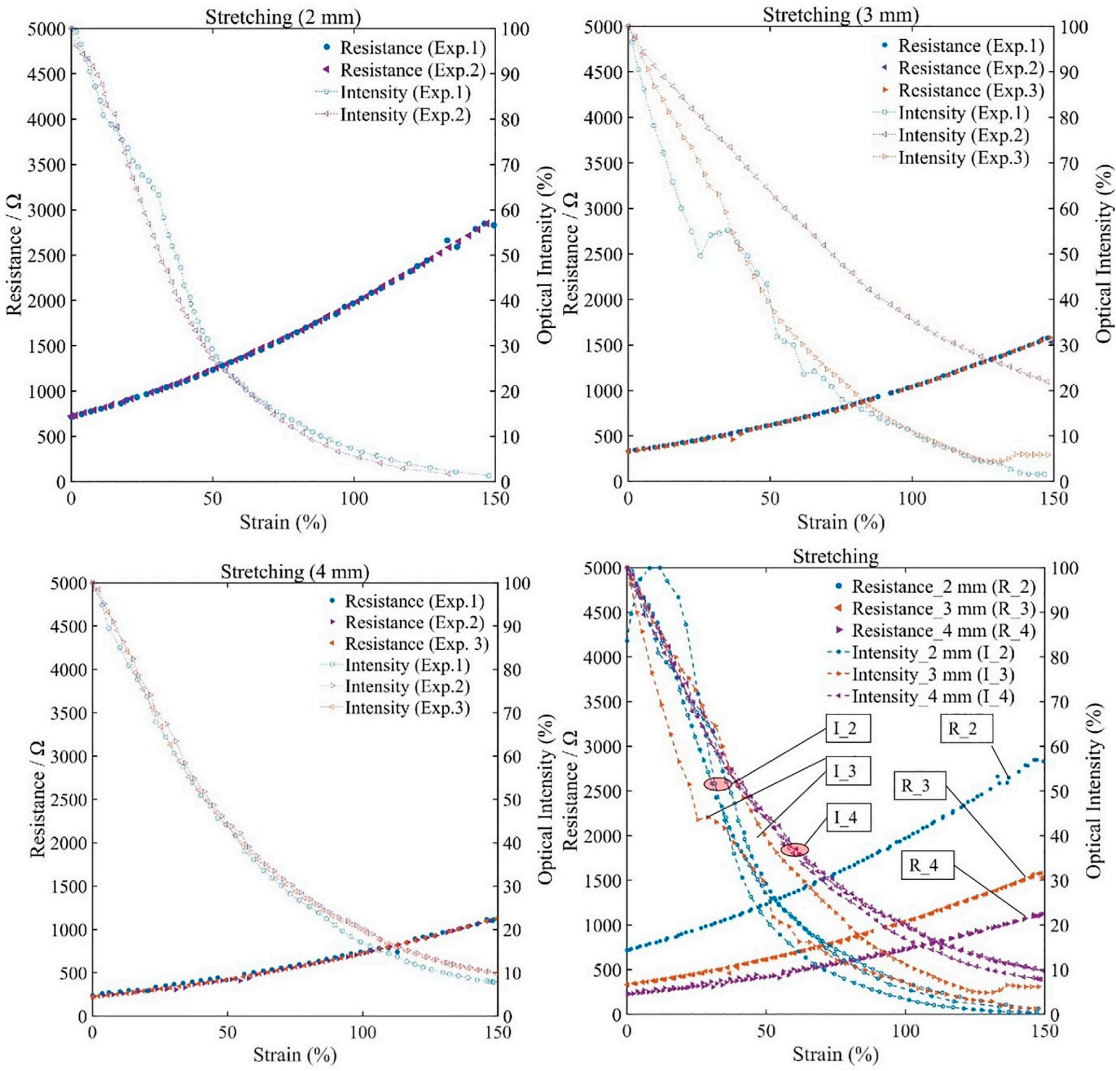
Resistance and optical intensity performances of the MHS Sensor variation during stretching. **(A)** Resistance and optical intensity variation of 2-mm-diameter sensor during stretching. **(B)** Resistance and optical intensity variation of 3-mm-diameter sensor during stretching. **(C)** Resistance and optical intensity variation of 4-mm-diameter sensor during stretching. **(D)** Comparison of performance of the MHS Sensor with three different diameters.

To validate the MHS Sensor’s capability of detecting deformation within large range, the stretching deformation was tested and both electrical and optical signals were collected. At room temperature (∼25°C), three groups of stretching tests were conducted with constant stepper motor speed, which means that the stretching was speed constant and continuous. The electrical and optical signals were outputted according to the strain by the aforementioned processing circuits. The testing results are shown in Figure 6. The resistances of the MHS Sensors at 0 strain increased with the aperture decrease (200, 300, and 700 Ω of 4, 3, and 2 mm respectively), agreeing with the material’s resistive characteristics. The optical intensity is expressed by the proportion to the maximum value measured, illustrating a relative intensity variation.

Highly repeatable varying processes were observed among different groups of experiments. Figure 6 illustrates the varying trend of the resistance and the optical intensity during stretching, respectively. The resistances increased (700 Ω/0-strain to 2800 Ω/150%-strain of 2-mm-diameter, 300 Ω/0-strain to 1500 Ω/150%-strain of 3-mm-diameter, and 200 Ω/0-strain to 1000 Ω/150%-strain of 4-mm-diameter) and optical intensities decreased (100%/0-strain to 0/150%-strain of 2 mm, 100%/0-strain to 0∼20%/150%-strain of 3 mm, and 100%/0-strain to 10%/150%-strain of 4 mm). The trend agrees well with the principle, since the area of cross section decreased and the length increased during stretching along the axis.

In addition, the results also show the remarkable stretching capability of the MHS Sensor that the strain could reach up to 150%. Such capability is realized by the hyper elastic silicone cladding, the strong adhesion, and the deformability of the hydrogel core.

Compared with the variations of the resistance, the optical intensity changes tend to be more obvious at the first 100% strain and less obvious afterward. This should be caused by that the optical intensity deteriorated to almost undetectable level at the distal end during the last 50% strain period. The MHS Sensor of a smaller diameter showed higher sensitivity of resistive variation while lower sensitivity of optical variation, leading to a selectable design strategy to balance the resistive and optical sensitivity by designating the appropriate diameter. With the validation of the resistance-strain and optical-strain relationships, the MHS Sensor’s features under stretching deformation could be analyzed.

### Twisting Tests

**FIGURE 7:**
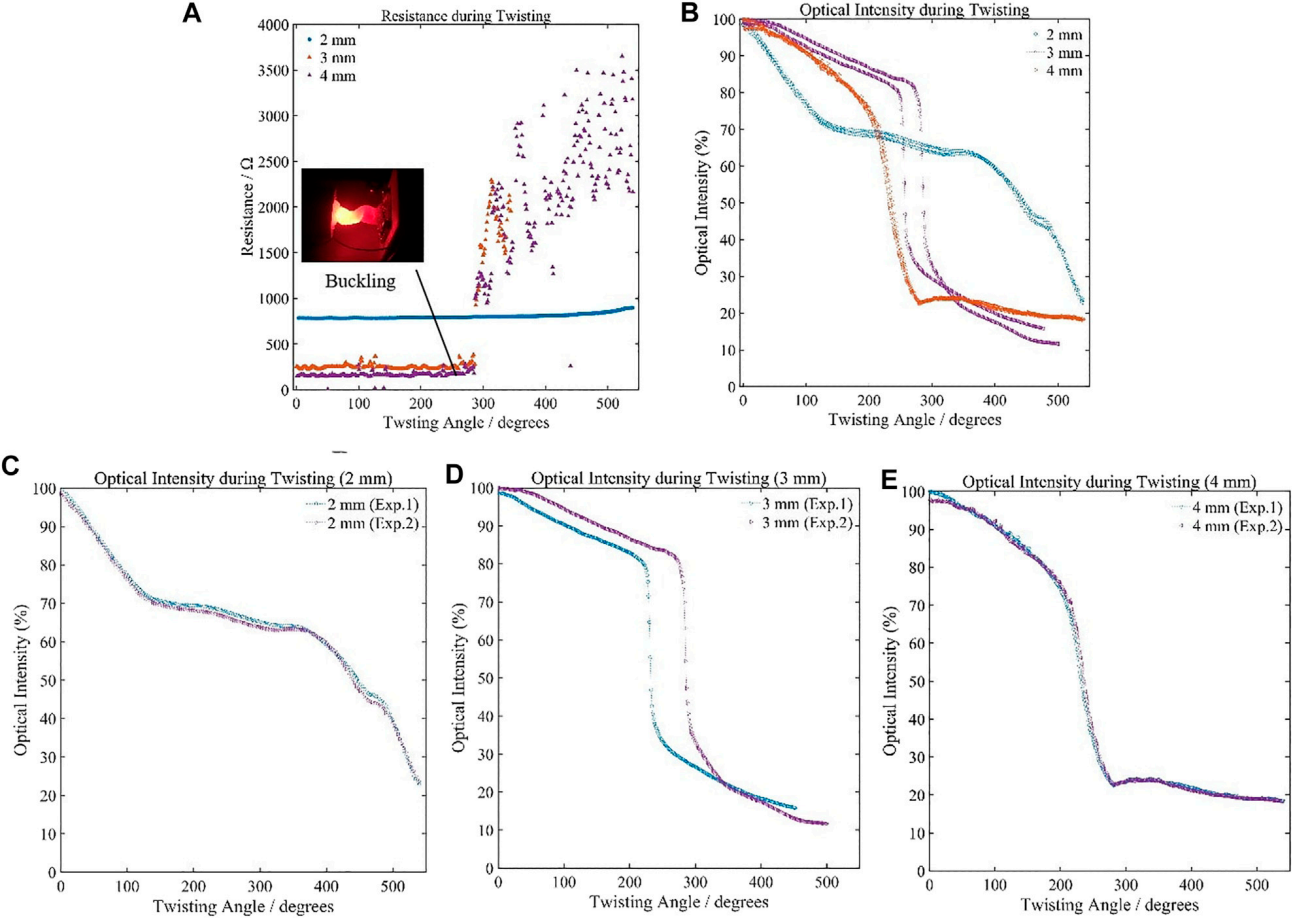
Resistance and optical intensity variation during twisting. **(A)** Sensing Resistance performances of the MHS Sensor with three different diameters. **(B)** Sensing optical intensity performances of the MHS Sensor with three different diameters. **(C)** Optical intensity variation of 2-mm-diameter sensor during twisting. **(D)** Optical intensity variation range of 3-mm-diameter sensor during twisting. **(E)** Optical intensity variation range of 4-mm-diameter sensor during twisting.

To investigate the relationship of the MHS Sensor’s properties under twisting, three groups of tests were conducted. At the room temperature, the sensor was twisted by the stepper motor with a constant speed from 0 to 540° (one and half circle), which was beyond the critical twisting angle once buckling happened. The results are shown in Figure 7. The resistance rarely changed during the whole twisting process while the optical intensity changed significantly. From the figure, the optical intensity could be observed a gradually decrease during the first 150° (100–75% of 2 mm), during the first 250° (100–70% of 3 mm, and 100–80% of 4 mm). Beyond 250°, the buckling began to happen and the aperture was nearly shut by the twisting deformation in 3 and 4 mm sensor. Therefore, the optical intensities decreased significantly, leading to the optical signal received by the photodiode deteriorating rapidly to the undetectable level. The 2 mm sensor didn’t buckle, which is also shown in the plot (Figure 7C). The MHS sensor, while being used under twisting deformation, will be limited within the nonbuckling range, where the application scenarios and specific diameter demand should be considered. The optical intensity and the twisting angle relationship indicates a higher sensitivity for the larger diameter sensors before buckling. Such a phenomenon agrees with the behaviors in stretching tests, that the geometrical parameters of the sensor would affect the sensitivity of the electrical and optical properties contrarily. The insight would guide the sensor design for the optimized performance according to the specific application scenarios.

### Repeatability and Duration Tests

**FIGURE 8:**
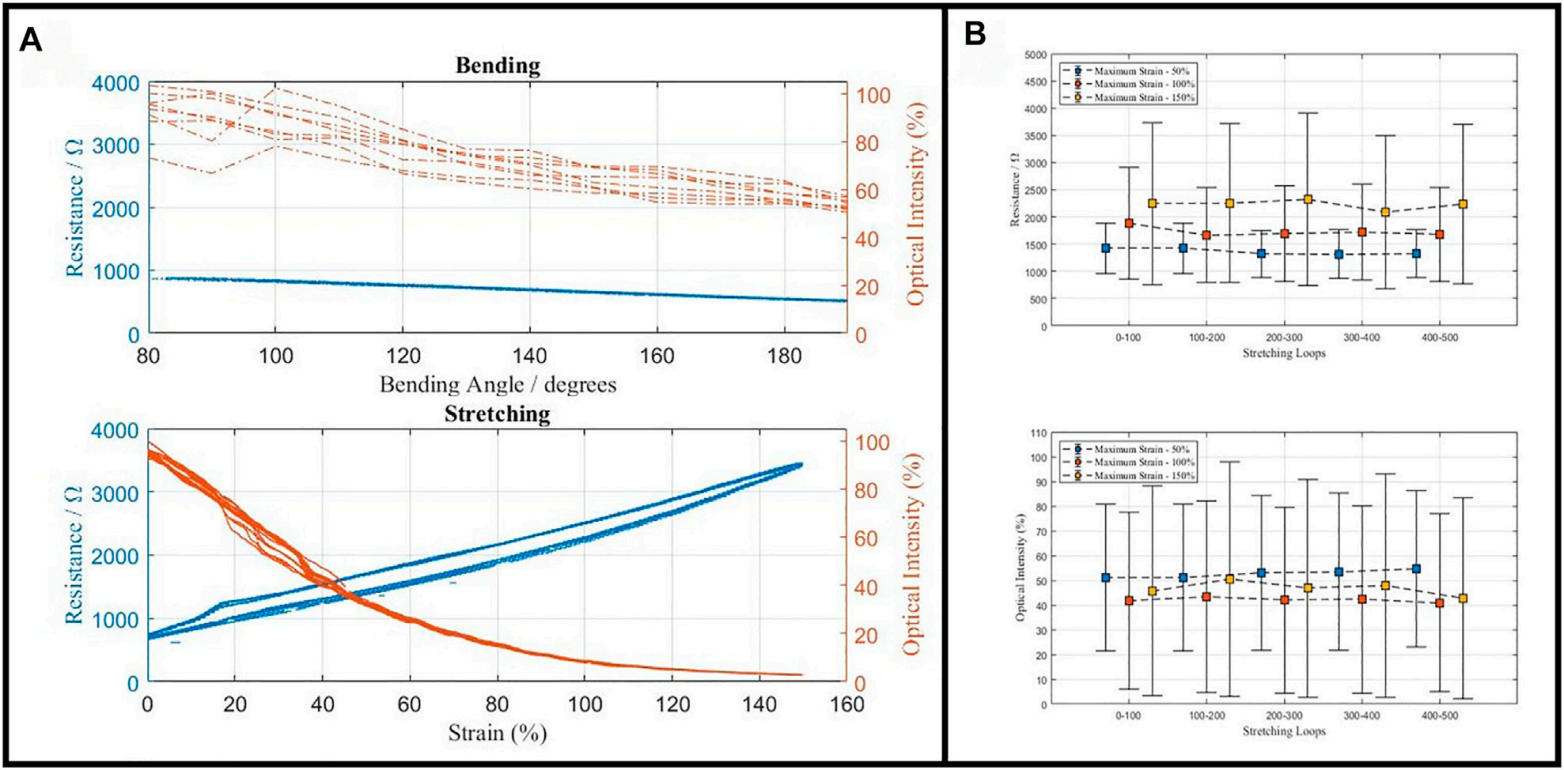
**(A)** The repetitive tests of the MHS sensor being bended, stretched, and twisted. **(B)** The duration tests of the MHS sensor, under 50, 100, and 150% stretching strain.

To validate the reproducibility of the experimental results, repetitive tests of the MHS sensor were conducted. In this part, stretching and bending loops of a fabricated prototype were done for eight times, respectively. Stretching repetitive tests were done with the range of 0–150% strain, and bending tests were from 70 to 140 bending angles. Resistance and optical signals were both collected. The results are shown in Figure 8A. For the bending of the MHS sensor, the resistance barely changed because the cross sectional area and the length of the sensor did not significantly change and the optical intensity decreased with the bending angle rising. Among the eight loops, from the results, the signal varying trends agreed well within the same testing group. The global maximum resistance deviation is 30 Ω and maximum optical intensity deviation is 10%. The most significant deviation happened in the range of 20–40 strain of stretching tests, which can be complemented by the resistance signal.

Apart from the repetitive tests, duration tests were conducted in terms of stretching. Each 500 cycles of 50% strain, 100% strain, and 150% strain were done collecting the resistance signals and optical signals, as shown in Figure 8B. One vertical bar shows one cycle of the stretching test, and the square shows the signal at the point of half strain of the corresponding cycle. Now big signal difference was observed after 500 cycles of test, indicating that the duration of the proposed MHS sensor can handle at least 500 usages under 150% strain.

### Humidity Tests

**FIGURE 9:**
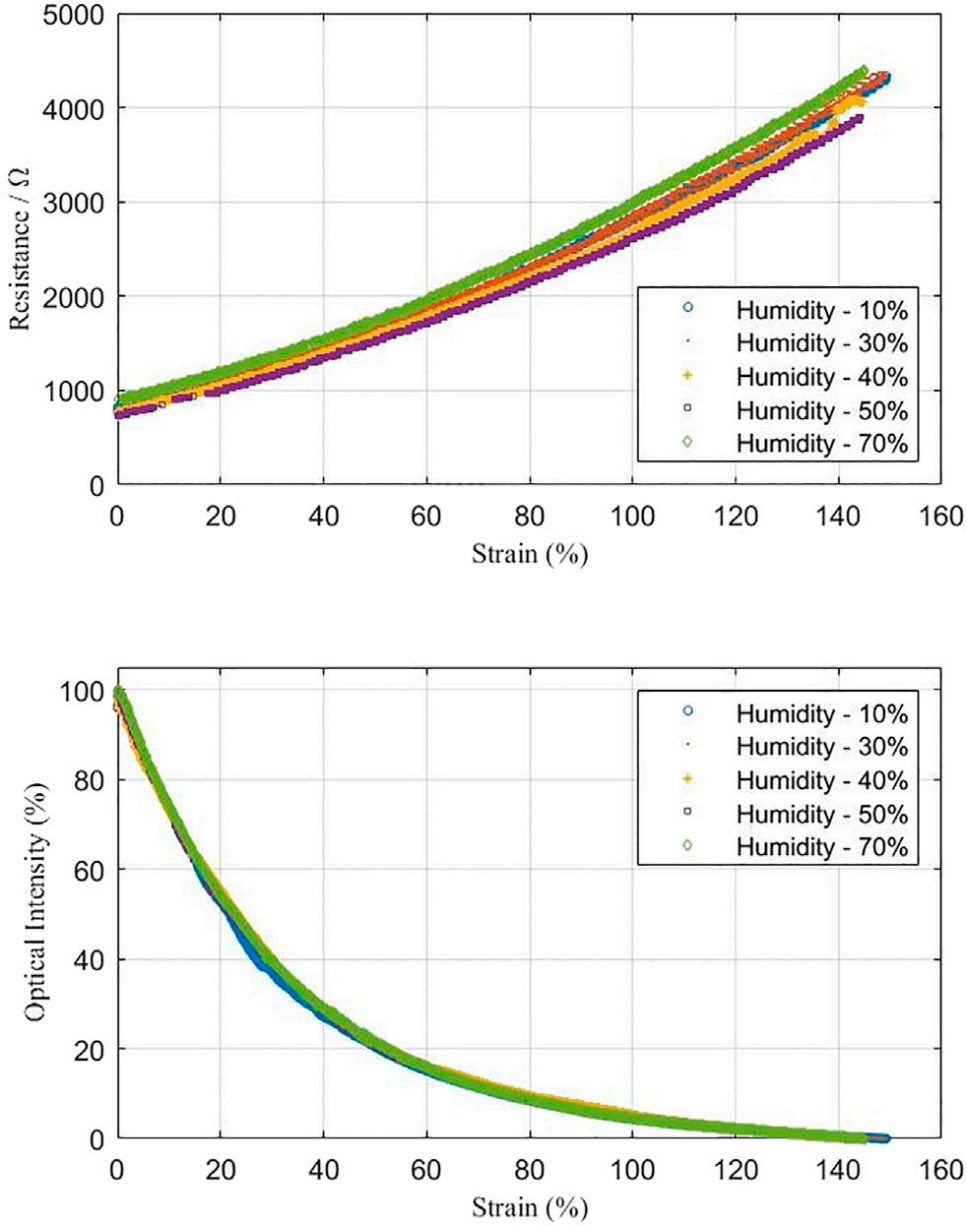
Stretching tests under different humidity situations, from 10 to 70% ambient humidity.

Although there is a silicone cladding to surround and protect the hydrogel inside, separating it with the environment, the dehydrating may affect the performance of the sensor. Therefore, the feasibility of the proposed sensor working in an environment with different humidity situations was tested. The results are shown in Figure 9. Seven sets of experiments were conducted, where 10, 20, 30, 40, 50, 60, and 70% humidity levels were set. Through the results, the resistance and optical intensity of the proposed sensor did not differentiate among seven groups, indicating that the silicone cladding separates the hydrogel well with the outer environments. Such separation can protect the hydrogel from extreme surroundings to ensure the normal signal collecting.

### Temperature Estimation

**FIGURE 10:**
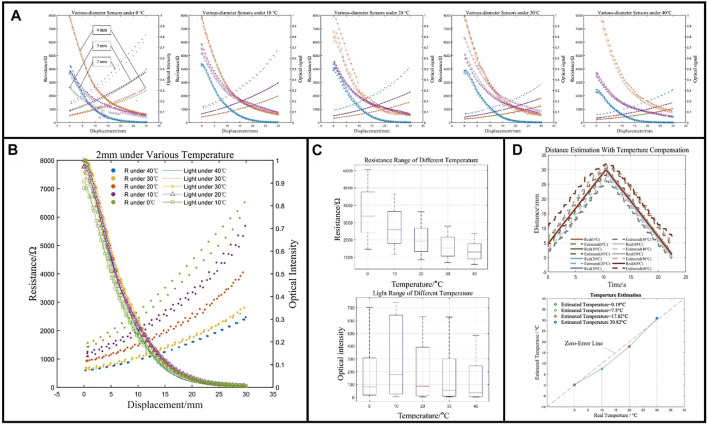
The performance of stretching under different ambient temperatures. **(A)** Sensing performances of the MHS Sensor with three different diameters. **(B)** Comparison of performance of 2-mm-diameter sensor with thermal stimulus. **(C)** Resistance and optical intensity variation range of 2-mm-diameter sensor with thermal stimulus. **(D)** Displacement estimation and ambient temperature estimation.

The sensors for soft robots are facing a critical application demand, which is operating in complex and harsh environmental factors. For instance, the ambient temperatures vary in a large range under water. To achieve multifunctional perception, the work further explored the performance of the MHS sensor at a variety of ambient temperatures. The incubator was adopted to generate a constant-temperature environment. Then the stretching tests of the three MHS Sensors with different diameters were conducted inside the incubator and the results are shown in Figure 10.

The hydrogel was first tested in a −18°C freezer for 2 h. No significant phrase change was obserbed, that the hydrogel still kept elastic and transparent under 0°Cwithout being frozen. From the results shown in Figure 10A, for each diameter MHS Sensor, the resistance of the hydrogel core decreased with the temperature increasing, with resistance under zero strain of 1500 Ω/0°C to 500 Ω/40°C of 2-mm-diameter, 1000 Ω/0°C to 300 Ω/40°C of 3-mm-diameter, and 500 Ω/0°C to 200 Ω/40°C of 4-mm-diameter. As the strain increasing, the resistance increased much slower at higher temperature than that at lower one, with the varying range of 5000 Ω/0°C to 2000 Ω/40°C of 2-mm-diameter, 3000 Ω/0°C to 1200 Ω/40°C of 3-mm-diameter, and 2000 Ω/0°C to 800 Ω/40°C of 4-mm-diameter.

In terms of the optical intensity, the larger diameter enhanced the light transmission to the photodiode and the diameter decrease caused by the stretching made the optical intensity deteriorate rapidly, illustrated by the earlier arrival of zero intensity of 2-mm-diameter sensor. Unlike the resistance characteristics, the optical intensity did not change with ambient temperatures (Figure 10B), 0.5/0mm to 0/30 mm of 2-mm-diameter, 0.6/0 mm to 0.1/30 mm of 3-mm-diameter, and 1.0/mm to 0.1/30 mm of 4-mm-diameter at arbitrary temperature, which means that the optical properties are insensitive to the temperature. From the results of 2-mm-diameter MHS Sensor (Figure 10C), the direct observation and comparison of the two signals show the varying electrical property and the constant optical property of the hydrogel under different ambient temperatures. Benefitting from such features, the optical signal could be the compensation when analyzing the stretching deformation with varying temperatures, shown by strain estimation in Figure 10D. This feature of the MHS Sensor also obtains the additional ability to calculate the temperature through reversely solving the signal–deformation relation.

The temperature perception could be achieved by finding temperature t which generates a least minimum square error of a linear model between the elongation of the sensor D, and the measured resistance of the sensor R,

$$\hat{t}=min\;\left(t,MS\;E\left(D=\left(\alpha t+k_{0}\right)R+\left(\beta t+k_{0}^{\prime }\right)\right)\right)$$ (7)

where D could be calculated using light signal of the experiment, $\alpha t+k_{0}\;$ stands for the sensitivity dependent on temperature, and $\beta t+R_{0}$ stands for the initial resistance also dependent on temperature. α,$k_{0},\beta$, and$R_{0}$ are constant terms that could be achieved by the experimental results. The estimation of the ambient temperature was shown in Figure 10D. The results show a minimum error of 0.19°C at 0°C and a maximum error of 2.5°C at 10°C, which verifies the ability of ambient temperature perception of the MHS Sensor.

### Pattern Recognition of the Deformation Modes

**FIGURE 11:**
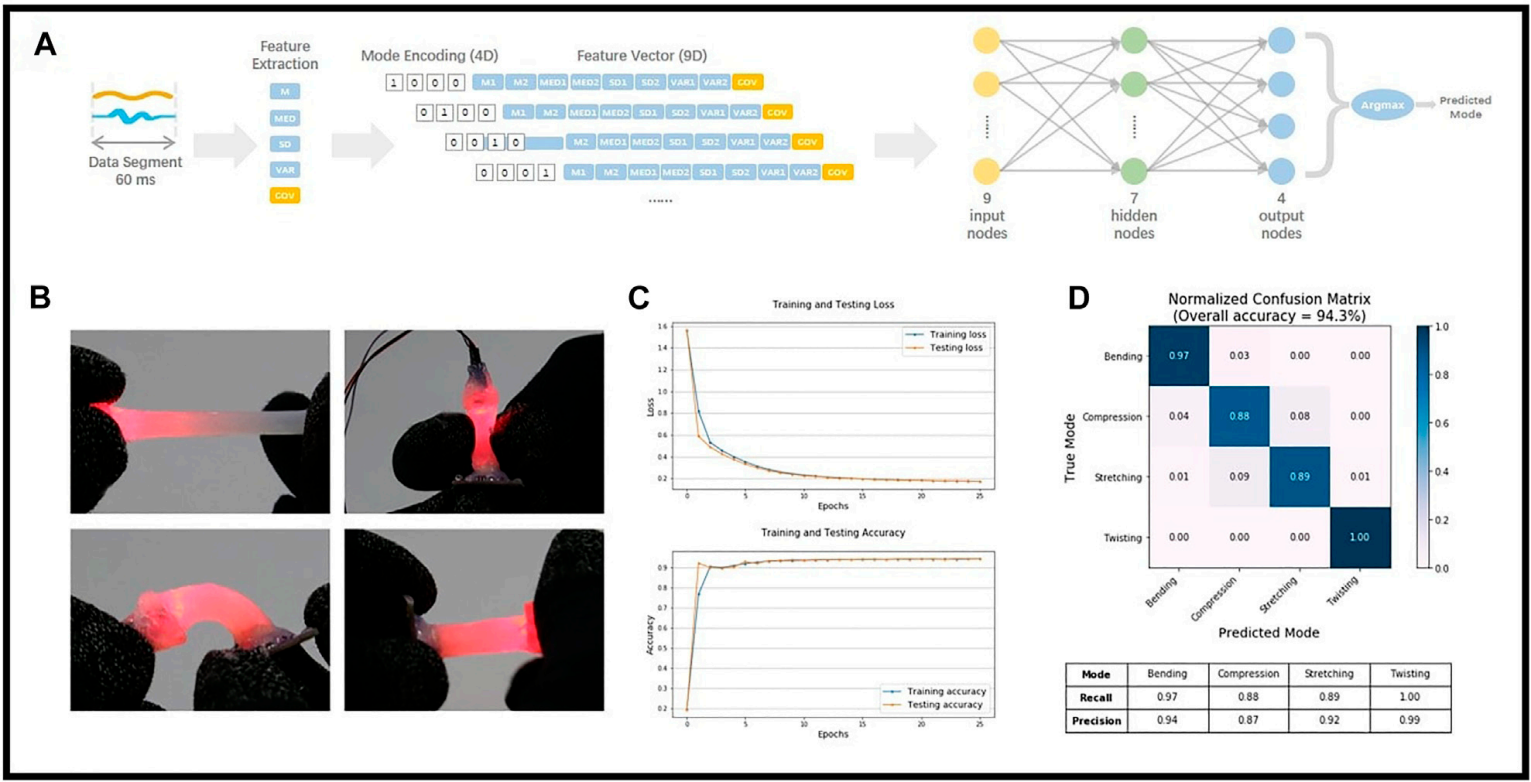
Deformation mode classification. **(A)** The classifier establishment. **(B)** Four passive movements: bending, compression, stretching, and twisting. **(C)** Model loss and accuracy. **(D)** Classification performance evaluation.

Benefit from the proposed dual-modality sensing mechanism, different deformation patterns of the MHS Sensor could be separated with the utilization of ANN. In this section, a simple classifier was trained to predict the deformation mode of the MHS Sensor with a dataset collected under four passive movements: bending, compression, stretching, and twisting (Figures 11A,B).

The continuous signals were split into 60-ms data segments with a 15-ms overlap between adjacent segments. The dataset was randomly divided into training dataset and testing dataset with a 90:10 split. 53,255 segments were used for the model training while 5,918 segments were only used for evaluating the classifier. Five features were extracted from each data segment. They are the Arithmetic Mean (M), Median (MED), Standard Deviation (SD) of the electrical and optical signals separately, and the Covariance (CDV) value between the two signals. A 9D feature vector was formatted eventually and constructed an input/output pair with a 4D label as the reference output. Accordingly, the light-weight ANN was built with a simple multilayer perceptron structure (9 input nodes, 7 hidden nodes, and 4 output nodes).

As Figure 11C shows, fast convergence of model loss was attained within 20 epochs. The fast-converging and lightweight classifier achieves a fair classification performance with a 94.3% overall accuracy on the testing dataset (Figure 11D). Especially, the mode twisting, which gets a 1.00 recall rate and 0.99 precision rate, could be recognized accurately among all the deformation modes.

Overall, through the experiments of stretching, twisting, and temperature, the sensing mechanism of the MHS Sensor could be validated and utilized after calibration. The experimental results and observations are of remarkable importance to the novel method of sensing deformations on robotics. The proposed soft sensor brought three main contributions: 1) substantially conforming to the large and complex deformation, being beneficial from its inherent elastic characteristics of both the cladding and the core; 2) being capable of sensing stretching and twisting with the integrated and compact design, utilizing both the electrical and optical properties variation when changing its geometrical parameters by deformation; 3) being able to compensate the thermal stimulus to the electrical properties of the hydrogel, meantime achieving the temperature estimation.

## Conclusion and Future Work

This work tackled the challenges in robotic sensing for large and complex deformation by offering a soft and multimodal approach to the sensing unit. The proposed MHS Sensor is entire solid state, and was investigated, showing its superior performance in large range stretching and twisting with the specific design. The MHS Sensor utilizes silicone molding and the hydrogel deploying techniques to provide a highly integrated and compact sensing system. Experiments were conducted on stretching, twisting platforms and in an incubator, revealing underlying mechanisms and distinctive characteristics of the proposed MHS Sensor. With such a design, the sensor could sense both proprio large and complex deformation and environmental temperature, achieving multifunctional perception. Furthermore, the deformation recognition was achieved by ANN with high accuracy.

This work also offers insights into robotic sensing system development with the solution to conform to and detect the coupled deformation, using property-change principles with multiple modalities. Moreover, the soft fabrication and hydrogellic approaches enable the capability of customization for sensor design due to varying demands of the specific applications. Such prodigious customized and individual unit could help design the whole robotic system more targetedly. In addition, the proposed sensor achieves extra perception within limited form factor, which could inspire novel ideas of compact sensor design. Such achievements pave the way for durable and compelling robotic sensing system in extreme application scenarios.

Future works include further investigation on more deformation modes, such as compression and coupled deformation. The concentration and the mechanical deformation brought by the temperature change should be investigated in a detailed manner. Furthermore, the adhesion between the hydrogel and the silicone cladding should be extra ensured with special techniques.

## Data Availability

The original contributions presented in the study are included in the article/Supplementary Material; further inquiries can be directed to the corresponding author.
